# TPX2 prompts mitotic survival via the induction of *BCL2L1* through YAP1 protein stabilization in human embryonic stem cells

**DOI:** 10.1038/s12276-022-00907-9

**Published:** 2023-01-04

**Authors:** Yun-Jeong Kim, Young-Hyun Go, Ho-Chang Jeong, Eun-Ji Kwon, Seong-Min Kim, Hyun Sub Cheong, Wantae Kim, Hyoung Doo Shin, Haeseung Lee, Hyuk-Jin Cha

**Affiliations:** 1 College of Pharmacy, Seoul National University, Seoul, 08826 Republic of Korea; 2 Department of Life Sciences, Sogang University, Seoul, 04107 Republic of Korea; 3 Drug Information Research Institute, College of Pharmacy, Sookmyung Women’s University, Seoul, 04310 Republic of Korea; 4 Department of Biochemistry, College of Natural Sciences, Chungnam National University, Daejeon, 34134 Republic of Korea; 5 College of Pharmacy, Pusan National University, Busan, 46241 Korea

**Keywords:** Embryonic germ cells, Apoptosis

## Abstract

Genetic alterations have been reported for decades in most human embryonic stem cells (hESCs). Survival advantage, a typical trait acquired during long-term in vitro culture, results from the induction of *BCL2L1* upon frequent copy number variation (CNV) at locus 20q11.21 and is one of the strongest candidates associated with genetic alterations that occur via escape from mitotic stress. However, the underlying mechanisms for *BCL2L1* induction remain unknown. Furthermore, abnormal mitosis and the survival advantage that frequently occur in late passage are associated with the expression of *BCL2L1*, which is in locus 20q11.21. In this study, we demonstrated that the expression of *TPX2*, a gene located in 20q11.21, led to *BCL2L1* induction and consequent survival traits under mitotic stress in isogenic pairs of hESCs and human induced pluripotent stem cells (iPSCs) with normal and 20q11.21 CNVs. High Aurora A kinase activity by TPX2 stabilized the YAP1 protein to induce YAP1-dependent *BCL2L1* expression. A chemical inhibitor of Aurora A kinase and knockdown of YAP/TAZ significantly abrogated the high tolerance to mitotic stress through *BCL2L1* suppression. These results suggest that the collective expression of *TPX2* and *BCL2L1* from CNV at loci 20q11.21 and a consequent increase in YAP1 signaling promote genome instability during long-term in vitro hESC culture.

## Stem cells: Maintaining stable cell cultures

New details of the molecular mechanisms behind problematic genetic aberrations that can affect cultured human embryonic stem cells could help efforts to maintain stable cell lines that hold great promise for treating a wide variety of diseases. Researchers in South Korea led by Hyuk-Jin Cha at Seoul National University investigated abnormalities in stem cell control systems leading to a condition known as survival advantage, which can allow abnormal cells to proliferate. They identified a gene (*TPX2*) whose expression activates another gene already known to be involved in triggering the survival advantage process. Their research further revealed that the protein encoded by *TPX2* achieves this effect indirectly, by interacting with another protein known to be able to control the activity of specific genes. Revealing this molecular signaling chain could assist culture of stable stem cells.

## Introduction

Due to their pluripotency, human embryonic stem cells (hESCs) have great potential in stem cell-based cell therapy; however, their frequent genetic aberrations during in vitro maintenance are considered a major hurdle that compromises the safety of this type of therapy^1,2^. However, despite these concerns^3^, few studies have explored the biological consequences, risks and reliable biomarkers of genetic alterations in hESCs^4^. As demonstrated by several extensive genomic studies, copy number variations (CNVs), including the 20q11.21 locus or trisomy of chromosome 12 or 17^5–7^, occur in hPSCs during in vitro culture. One of the most characteristic phenotypic changes of genetically aberrant hESCs is acquired culture adaptation^5^ for hESC survival under various stress conditions, including culture, genotoxic stress, and single-cell dissociation among others (also referred to as survival advantage)^8,9^. Such acquired survival traits result from the induction of *BCL2L1* (encoding BCL-xL), a typical antiapoptotic gene located at 20q11.21^8,10^ due to amplification of 20q11.21^11^ and/or dominant mutation of p53^12^. These alterations impair the major cell death mechanism of hESCs, as the mitochondria are primed to undergo cell death upon genotoxic stress through p53 translocation^13–16^. A recent study revealed that escape from mitotic cell death during mitotic errors due to this acquired survival trait (via either induction of *BCL2L1* or dominant mutation of *NOXA*, a proapoptotic factor) leads to aneuploidy^9^. In addition, a targeting protein for Xklp2 (TPX2), also located in 20q11.21, was suggested to be the putative driver of abnormal mitosis by disrupting spindle dynamics^17^. However, despite the significance of survival advantage in the phenotypic changes of culture-adapted hESCs and even aneuploidy, the molecular mechanisms underlying this phenomenon, with the exception of *BCL2L1* induction, are not fully understood. Moreover, a reduction in serum response factor expression^18^ or high oxygen concentrations during in vitro culture^19^ have been associated with hESC genetic alterations.

High susceptibility to genotoxic stress in hESCs is a unique cellular characteristic that has developed as a safeguard for genome integrity^20^. However, the contemporaneous expression of antiapoptotic factors maintains a fine balance between survival and apoptosis^21^. It is also worth noting that deletion of Yap1 leads to embryonic lethality^22^, suggesting that Yap1 is required for the self-renewal and differentiation of mouse ESCs (mESCs)^23^. A recent study demonstrated that Yap1 in mESCs serves as a safeguard to attenuate mitochondrial apoptosis by upregulating typical antiapoptotic factors, including *Bcl2l1*^24^. Similarly, Rho-dependent activation of YAP1 promotes the long-term survival of hESCs^25^. In addition to ESCs, YAP1 activation in cancer cells increases survival through the induction of *BIRC5* and *BCL2L1*, both of which are important for hESC survival^26^.

In this study, using four types of isogenic hESCs after different periods of in vitro culture^8^ and with different CNV statuses, we demonstrated that *TPX2*, located at locus 20q11.21, was highly induced in culture-adapted hESCs and conferred resistance under mitotic stress through *BCL2L1* induction. Our findings also revealed that the YAP1 protein stabilization conferred by Aurora A kinase activated by *TPX2* induction was responsible for *BCL2L1* transcription, resulting in mitotic stress escape and suggesting that additional signaling (e.g., YAP1) would be required for determining culture-adapted phenotypic changes in hESCs other than CNVs.

## Materials and methods

### Reagents and cell culture

The primary antibodies (Supplementary Table 1) and chemical reagents (Supplementary Table 2) are listed. Human embryonic stem cells (WA09: H9, WiCell Research Institute, CHA3-hESCs) were maintained as previously described^8^. Medium and reagents for cell culture are listed in Supplementary Table 3. Sequence information of RT-PCR (Supplementary Table 4) and siRNA (Supplementary Table 5) is listed.

### Genome-wide gene expression profiling

Assessment of the differences in gene expression between groups was conducted using the R package DESeq2 from the FASTQ files and processed data in the Gene Expression Omnibus (GEO: GSE167495). Differently expressed genes (DEGs) were selected with |log_2_fold-change | >1 and a false discovery rate (FDR) of <0.01. To identify genomic loci containing the most DEGs, overrepresentation analysis was performed using a hypergeometric test with the positional gene sets from the Molecular Signatures Database (MSigDB, https://www.gsea-msigdb.org/gsea/msigdb/). After FDR-based multiple testing correction, an enrichment score for each genomic locus was defined as the -log_10_ Q-value. For GO analysis, GSEA was performed using the GO BP gene sets from MSigDB via the R package fgsea. Significantly upregulated or downregulated GO terms were selected with an adjusted *P* value of <0.01 and a |normalized enrichment score (NES) | of >2.

### Determination of copy number variations

Whole-genome genotyping was performed using the Illumina HumanOmni1-Quad Beadchip (Illumina) containing 1,140,419 genetic markers across the human genome. Samples were processed according to the specifications of the Illumina Infinium HD super assay. Briefly, each sample was whole-genome amplified, fragmented, precipitated, and resuspended in an appropriate hybridization buffer. Denatured samples were hybridized on a prepared BeadChip for a minimum of 16 h at 48 °C. Following hybridization, the bead chips were processed for the single-base extension reaction, stained, and imaged with an Illumina iScan system. Normalized bead intensity data for each sample were loaded into the GenomeStudio software package (Illumina). Signal intensity ratios were calculated using the log R ratio (LRR: log ratio of observed probe intensity to expected intensity; any deviations from zero in this metric are evidence of copy number change), and allelic intensity was determined by the B allele frequency for all samples. Values were exported using Illumina GenomeStudio. Structural variant analysis was performed using the sliding window approach (window size 10).

### Live cell imaging

Live cell images were captured by JuLI^TM^ Stage (NanoEnTek Inc.). The acquired images were further processed and analyzed with JuLI^TM^STAT software according to the manufacturer’s instructions.

### Statistical analysis

Graphical data are presented as the mean ± S.E.M. Statistical significance for more than three groups was determined using one-way or two-way analysis of variance (ANOVA) followed by a Tukey multiple comparison posttest. Statistical significance between two groups was analyzed using unpaired Student’s *t* tests. Statistical analysis was performed with GraphPad Prism 8 software (https://www.graphpad.com/scientific-software/prism/). Significance was assumed for *p* < 0.05 (*), *p* < 0.01 (**), and *p* < 0.001 (***).

### Supplementary data

Supplementary data are available at EMM online.

## Results

### The survival advantage of culture-adapted hESCs under mitotic stress

**Fig. 1:**
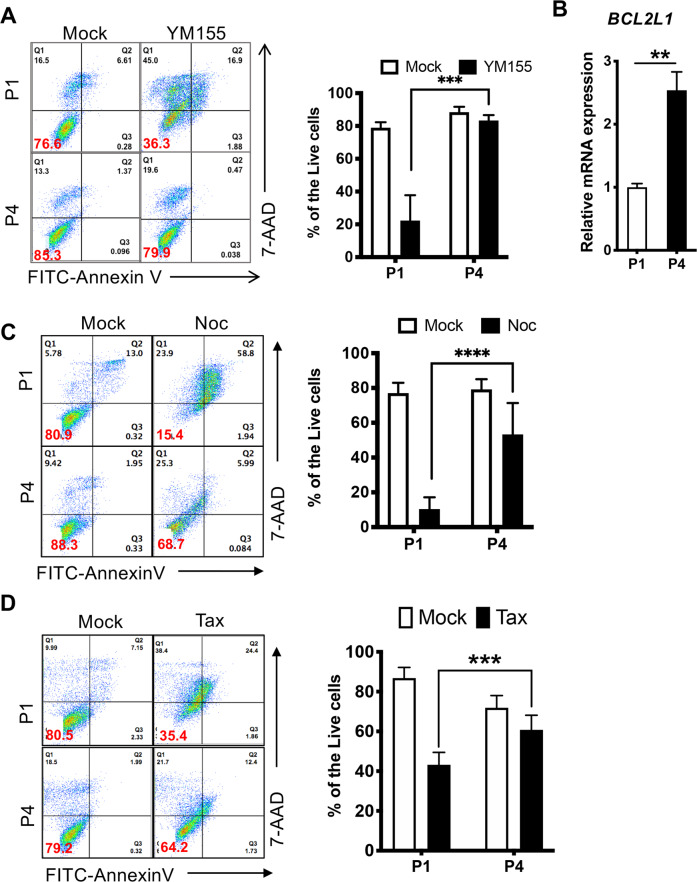
The survival advantage of culture-adapted hESCs under mitotic stress. **A** After treatment with YM155 (50 nM) for 24 h, the death of P1 and P4 hESCs was determined by Annexin V/7-AAD staining (*n* = 6 independent experiments; mean ± SEM, two-way ANOVA, ***p < 0.001). **B** Relative mRNA expression level of *BCL2L1* in P1 and P4 hESCs (*n* = 5 independent biological repeats; independent experiments; mean ± SEM, Mann‒Whitney test, **p < 0.01). **C**, **D** Death of P1 and P4 hESCs after treatment with 50 ng/ml nocodazole (Noc: **C**) or 50 ng/ml paclitaxel (Tax: **D**) measured by flow cytometry and stained with Annexin V and 7-AAD (*n* = 5 independent experiments; mean ± SEM, two-way ANOVA, ****p < 0.0001 for **C**; *n* = 6 independent experiments, two-way ANOVA, ***p < 0.001 for **D**).

Culture-adapted hESCs become highly resistant to various stressors, such as DNA damage^10^, single-cell dissociation, and YM155^8^, ‘a stemtoxic agent’ that selectively induces death in undifferentiated pluripotent stem cells^13^ through cellular uptake by SLC35F2, a solute carrier protein^27,28^. In addition to the increased survival of hESCs under genotoxic stress, escape from cell death during mitosis has been suggested to increase aneuploidy during long-term culture^9^. To examine the effect of the survival trait acquired in long-term in vitro culture, we took advantage of a unique isogenic set of hESCs after different periods of in vitro culture^8,17^ [e.g., P1 hESCs: fewer than 50 passages (1 year of culture); P2 hESCs: over 100 passages (2 years of culture); P3 hESCs: over 200 passages (4 years of culture); and P4 hESCs: over 300 passages (over 6 years of culture)]. As described previously^8,17^, a clear survival trait after YM155 treatment was evident in P4 hESCs compared to P1 hESCs (Fig. 1A) and was associated with *BCL2L1* induction (Fig. 1B). Next, to examine the escape from mitotic cell death induced by mitotic stresses, which was previously suggested to be a possible cause of aneuploidy in hESCs^9^, P1 or P4 hESCs were subjected to mitotic stress with typical drugs targeting mitotic spindles such as nocodazole (Noc: a spindle destabilizer) and paclitaxel (Tax: a spindle stabilizer). Similar to YM155 treatment (Supplementary Fig. 1a) and single-cell dissociation (Supplementary Fig. 1b), P4 hESCs, which display acquired survival traits^8^ as well as abnormal mitosis^17^, were highly resistant to mitotic stress inducers (Fig. 1C and D).

Except for the role of *BCL2L1* induction followed by CNV at locus 20q11.21^11^ or p53 dominant mutations^12^, little is known regarding the mechanisms by which the survival trait is acquired during hESC culture adaptation. Furthermore, no previous studies have determined how *BCL2L1* transcription becomes upregulated, resulting in the survival advantage trait in culture-adapted hESCs. We observed that the survival trait manifested in P3 hESCs (over 200 passages) and P4 hESCs (over 300 passages) but not P2 hESCs (over 100 passages) under either YM155 treatment (Supplementary Fig. 1a), cell dissociation (Supplementary Fig. 1b) or mitotic stress (Supplementary Fig. 1c), with *BCL2L1* expression comparable to that of P1 hESCs (less than 50 passages)^8^.

### *BCL2L1* induces survival associated with TPX2

**Fig. 2:**
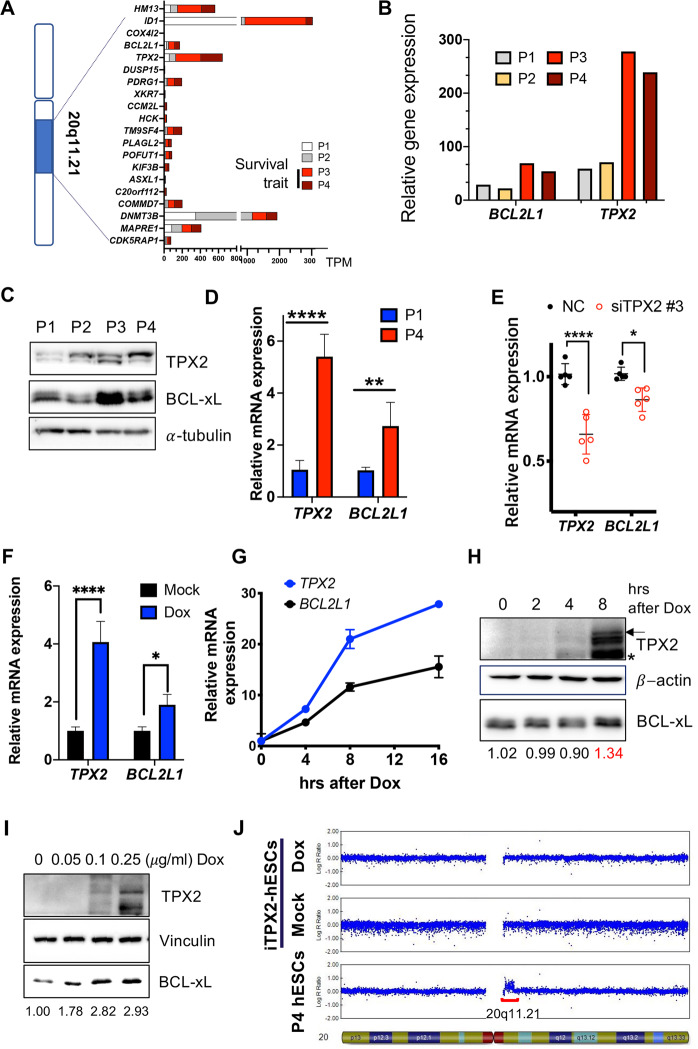
*BCL2L1* induces survival associated with TPX2. **A** Graphical representation of chromosome 20 and the 20q11.21 locus with the accompanying genes (left) and relative expression levels of the genes at the 20q11.21 locus in P1, P2, P3, and P4 hESCs (right). **B** Relative transcript levels of *BCL2L1* and *TPX2* in P1, P2, P3, and P4 hESCs. **C** Immunoblotting for TPX2 and BCL-xL from the indicated hESCs; α-tubulin was utilized for equal protein loading. **D** The relative mRNA expression level of the indicated gene in P1 and P4 hESCs (*n* = 7 independent biological repeats; mean ± SEM, Mann‒Whitney test, **p < 0.01, *p < 0.05). **E** Relative mRNA expression levels of *TPX2* and *BCL2L1* in P4 hESCs 48 h after siRNA administration in the control (NC) and TPX2 groups (siTPX2#3) (*n* = 5 independent experiments; mean ± SEM, two-way ANOVA, ****p < 0.0001, *p < 0.05). **F** Relative mRNA expression levels of *TPX2* and *BCL2L1* in H9-iTPX2-hESCs 24 h after treatment with 0.1 μg/ml Dox (*n* = 4 independent experiments, mean ± SEM, two-way ANOVA, ****p < 0.0001, *p < 0.05). **G** Relative mRNA expression levels of *TPX2* and *BCL2L1* in iTPX2-hESCs at the indicated times after treatment with 0.1 μg/ml Dox. **H** Immunoblotting of TPX2 and BCL-xL in iTPX2-hESCs at the indicated time after treatment with 0.1 μg/ml Dox (arrow for induced GFP-TPX2, * for endogenous TPX2). Relative band densities of BCL-xL are shown. **I** Immunoblotting of TPX2 and BCL-xL in iTPX2-hESCs 24 h after treatment with the indicated dose of Dox. Relative band densities of BCL-xL are shown. **J** Copy number output using the Illumina Asian Screening Array (~700 K) computed by GenomeStudio software provided by Illumina. The two plots shown are for B allele frequency and log R ratio. The copy number gain on chromosome 20 detected in H9-P4 hESCs based on the log R ratio is shown by a red bar.

As previously demonstrated, gain of 20q11.21 was the most frequently occurring genome aberration and CNV of *ID1, BCL2L1*, and *HM13* at locus 20q11.21, suggesting that the expression of these genes was a marker of genome aberration^5^ and may lead to abnormal biological consequences^10,11,29^. However, intriguingly, not all genes at locus 20q11.21 were transcriptionally active in hESCs with 20q11.21 CNV (e.g., P3 and P4 hESCs) (Fig. 2A). Among the genes in the 20q11.21 locus, TPX2, which was previously shown to induce aberrant mitosis in culture-adapted hESCs^17^, was concurrently induced with *BCL2L1* in P3 and P4 hESCs (Fig. 2B). Another pair of human pluripotent stem cells (hPSCs) at early and late passages (e.g., hCHA3 and BJ-iPSCs), of which the survival trait as well as trisomy at chromosome 12 was manifested in late-passage hPSCs [e.g., passage 181 (P181) for BJ-iPSCs and passage 333 (P333) for CHA3]^8^, also revealed that hPSCs with gain of 20q11.21 (Supplementary Fig. 1d) at the late passage expressed high levels of *TPX2* and *BCL2L1* (Supplementary Fig. 1e). Similarly, the BCL-xL protein level was closely associated with the TPX2 protein level in P3 and P4 hESCs, where the survival trait was evident (Fig. 2C). Notably, P4 hESCs with high TPX2 expression also exhibited higher levels of *BCL2L1* (Fig. 2D). Considering the roles of TPX2 in cancer malignancy^30^ and survival/chemoresistance^31^, it was readily presumed that TPX2 expression may be associated with *BCL2L1* induction and the consequent survival traits. As predicted, partial depletion of TPX2 in P4 hESCs (Fig. 2E) and P3 hESCs (Supplementary Fig. 2a) significantly attenuated *BCL2L1* expression. Due to the significantly high *BCL2L1* and TPX2 coexpression in P3 and P4 hESCs validated by multiple reference genes (Supplementary Fig. 2b and c), alteration of *BCL2L1* followed by TPX2 depletion suggests that TPX2 induction somehow regulates *BCL2L1* expression and confers the survival trait. Of note, due to the pivotal roles of TPX2 in not only spindle integrity and genome stability but also early embryogenesis^32^, the establishment of stable TPX2 knockdown in P4 hESCs or TPX2 expression in P1 hESCs was unsuccessful despite multiple trials (data not shown). Thus, alternatively, the doxycycline (Dox)-inducible TPX2 cell line established from P1 hESCs (iTPX2-hESCs) was used to explore the role of *TPX2* in *BCL2L1* induction (Supplementary Fig. 2d). TPX2 mRNA (Supplementary Fig. 2d) and protein (Supplementary Fig. 2e) induction occurred in a dose-dependent manner. The signal from TPX2 conjugated with green fluorescent protein (GFP) was evident in the mitotic spindle, where TPX2 is located during mitosis^33^ (Supplementary Fig. 2f). Surprisingly, TPX2 induction was sufficient to increase *BCL2L1* mRNA (Fig. 2F and G) and protein expression in a time- (Fig. 2H) and dose-dependent manner (Fig. 2I). It is important to note that iTPX2-hESCs were established from P1 hESCs with normal copy numbers, and therefore, the copy number of iTPX2-hESCs remained normal regardless of Dox treatment, unlike that observed in P4 hESCs (Fig. 2J). The regulatory effect of TPX2 on *BCL2L1* was reproduced in iTPX2-hESCs derived from hCHA3 hESCs with consistent results (Supplementary Fig. 2g).

### TPX2 induction rescues mitotic cell death of normal hESCs

**Fig. 3:**
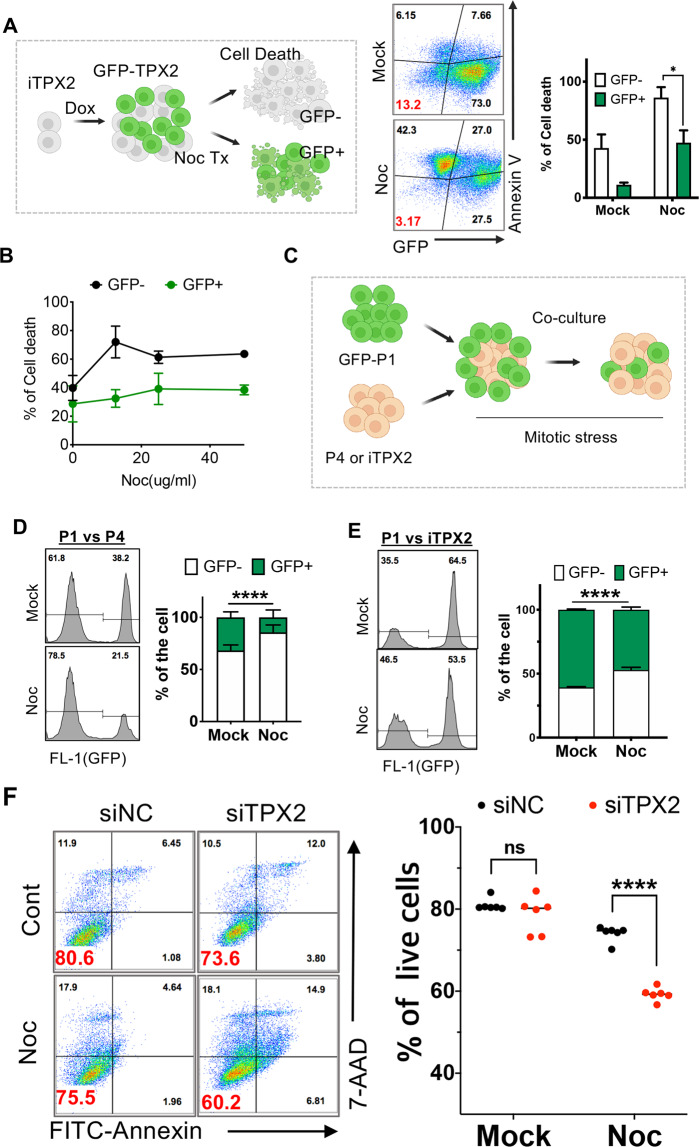
TPX2 induction rescues mitotic cell death in normal hESCs. **A** Graphical representation of the effect of TPX2 induction on cell death after Noc treatment in hESC-iTPX2 (left) and flow cytometry analysis of PE-Annexin V staining after iTPX2 hESCs were pretreated with 0.1 μg/ml Dox or DMSO (Mock) for 16 h in the presence or absence of nocodazole (50 ng/ml) (Noc Tx). The bar graph shows the death (%) of GFP-negative and GFP-positive cells (right) (*n* = 4 independent experiments; mean ± SEM, Mann‒Whitney test, *p < 0.05). **B** Graph of % of cell death determined by 7-AAD staining of the GFP-negative or GFP-positive population after administration of the indicated dose of Noc. **C** Graphical presentation of the competition assay with coculture of the indicated cell types. **D**, **E** Flow cytometry analysis of P1 and P4 hESCs (**D**, *n* = 6 independent experiments; mean ± SEM, Two-Way ANOVA, ****p < 0.0001) or P1 and iTPX2 hESCs (**E**, *n* = 5, two-way ANOVA, *p* value < 0.0001) after treatment with 50 ng/ml of Nocodazole for 24 h. Populations were identified by GFP after 2 days of Noc treatment. **F** Flow cytometry analysis of 7-AAD/Annexin V staining of P4 hESCs after control (siNC) or TPX2 siRNA (siTPX2) treatment (*n* = 6 independent experiments; mean ± SEM, two-way ANOVA, ****p < 0.0001).

We previously demonstrated that TPX2, located at locus 20q11.21 near *BCL2L1*, is a putative driver of abnormal mitosis^17^. Using iTPX2-hESCs, cell death under mitotic stress (i.e., Noc) was determined after TPX2 induction by Dox. We noted that only a portion of the cells expressed GFP-TPX2 even after Dox treatment for unknown reasons. Thus, a GFP-negative population was used as an internal control, and cell death among the GFP-positive population (expressing TPX2) of iTPX2-hESCs (Supplementary Fig. 3a) was monitored after mitotic stress (Fig. 3A, left panel). As predicted, GFP-positive cells were more resistant to mitotic stress induced by Noc (Fig. 3A, right panel) than GFP-negative cells. Similar results were obtained at different doses of Noc (Fig. 3B). Resistance to two types of mitotic stress inducers via TPX2 induction was reproduced in other hCHA3-derived iTPX2-hESCs (Supplementary Fig. 3b and c). To rule out the potential prosurvival effect of GFP expression (rather than the effect of TPX2), P1 hESCs expressing enhanced green fluorescent protein (EGFP-P1) were cocultured with P4 hESCs and iTPX2 hESCs (Fig. 3C), as the basal TPX2 levels in P4 hESCs and iTPX2 hESCs [due to leakage of the Tet-O system^34^] were comparably higher than that in P1 hESCs (Supplementary Fig. 3d). Consistently, hESCs with high TPX2 expression (e.g., P4 and iTPX2) were more resistant to Noc-induced mitotic stress regardless of GFP expression (Fig. 3D and E). To further assess whether high TPX2 expression was responsible for mitotic stress resistance, TPX2 was transiently depleted by multiple siRNAs. P4 hESCs with TPX2 depleted to a level similar to that in P1 hESCs (Supplementary Fig. 3e) sensitized the cells to Noc-induced mitotic stress (Fig. 3F). In addition, TPX2 depletion with independent siRNA (Supplementary Fig. 3f) sensitized P4 hESCs to Noc-induced cell death (Supplementary Fig. 3g).

### YAP/TEAD4 leads to *BCL2L1* expression

**Fig. 4:**
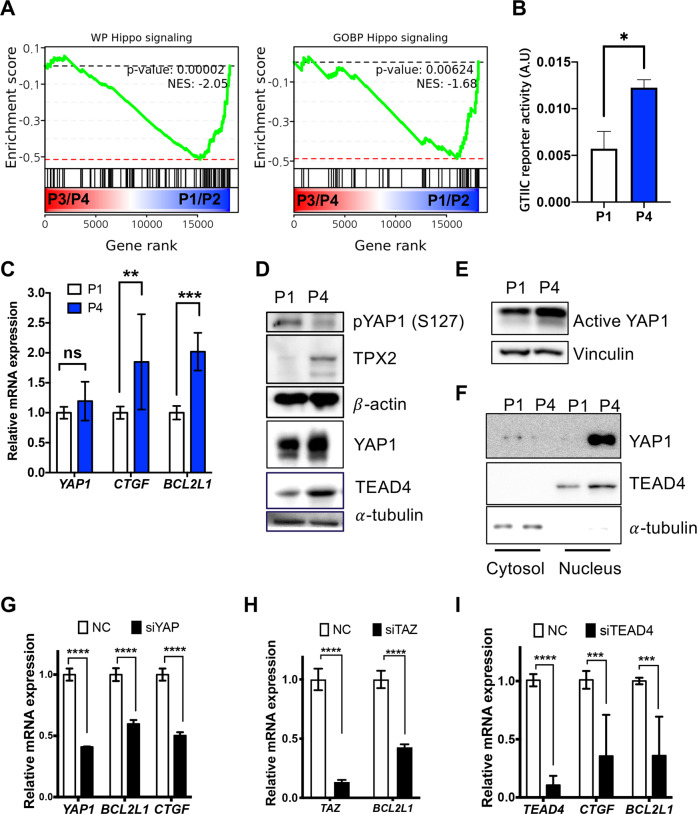
YAP/TEAD4 leads to *BCL2L1* expression. **A** GSEA of WP_PATHWAYS_REGULATING_HIPPO_SIGANLING (left) and GOBP_REGULATION_OF_HIPPO_SIGNALING (right) of P1/P2 hESCs and P3/P4 hESCs from the RNA-seq data of P1, P2, P3 and P4 hESCs (GSE167495). **B** 8X GTIIC reporter assay of P1 and P4 hESCs (*n* = 4 independent experiments; mean ± SEM, Mann–Whitney test, *p < 0.05). **C** Relative mRNA levels of YAP1, CTGF and *BCL2L1* in P1 or P4 hESCs (*n* = 6 independent experiments; mean ± SEM, two-way ANOVA, **p < 0.01, ***p < 0.001). **D** Immunoblotting of TPX2, YAP1, TEAD4 and phosphorylation of YAP1 (at serine 127, S127: α-tubulin or β-actin were utilized for equal protein loading. **E** Immunoblotting for active YAP1 in P1 and P4 hESCs. Vinculin was used as a loading control. **F** Immunoblotting for YAP1 and TEAD4 in the cytosol or nuclei of P1 or P4 hESCs., α-Tubulin was used for equal protein loading. **G**–**I** Relative mRNA expression of the indicated genes in P4 hESCs after control (NC) or siRNA for (**G**) YAP1 (*n* = 3 independent experiments; mean ± SEM, two-way ANOVA, ****p < 0.0001) (**H**) TAZ (*n* = 4 independent experiments; mean ± SEM, two-way ANOVA, ****p < 0.0001), or (**I**) TEAD4 (*n* = 5 independent experiments; mean ± SEM, two-way ANOVA, ***p < 0.001, ****p < 0.0001).

*BCL2L1*, an antiapoptotic factor highly expressed in P4 hESCs (Fig. 2D), has been shown to be a downstream target of YAP1 in cancer cell models^26^. Considering the key roles of YAP1 in the survival of ESCs^24,25^, we hypothesized that the activity of YAP1 may account for the acquisition of survival traits in culture-adapted hESCs. It has been previously demonstrated that Rho and Hippo regulation of YAP/TAZ activation is critical for the survival response of hESCs^25^. Similarly, two independent gene sets for Hippo signaling were enriched in P1 and P2 hESCs (showing cell death susceptibility) but produced low signaling in P3 and P4 hESCs (showing the survival trait) based on Gene Set Enrichment Analysis (GSEA) (Fig. 4A and Supplementary Fig. 4a). Moreover, YAP1 activity determined by GTIIC reporters was significantly higher in P4 hESCs (Fig. 4B). Considering the pivotal role of YAP1 in the survival of both mouse and human ESCs^24,25^, the high YAP1 activity acquired during in vitro culture could further enhance the survival signal, thus resulting in a survival advantage. Although the YAP1 mRNA level itself remained unaltered, the *BCL2L1* mRNA level was higher in addition to CTGF [encoded by *CCN2*, a typical YAP1 downstream target gene^35,36^] in P4 hESCs than in P1 hESCs (Fig. 4C). Despite similarities in YAP1 transcription, the YAP1 protein level was apparently stabilized with lower amounts of phosphorylated YAP1 (at S127) in P4 hESCs with higher TPX2 and TEAD4 expression (Fig. 4D), a finding that was further validated by an anti-active YAP antibody detecting levels of unphosphorylated YAP^37^(Fig. 4E). Consistently, nuclear YAP1 expression manifested along with TEAD4 protein expression in P4 hESCs (Fig. 4F). In contrast, depletion of YAP1 (Fig. 4G), TAZ (Fig. 4H), or TEAD4 (Fig. 4I) significantly decreased *BCL2L1* expression along with CTGF in P4 hESCs. The same result was obtained with different sets of reference genes (Supplementary Fig. 4b). The other sets of hPSCs (i.e., BJ-iPSCs and CHA3) showing survival traits with gain of 20q11.21 (i.e., P181 for BJ-iPSCs and P333 for CHA3) expressed high levels of TPX2 and BCL-xL, corresponding to high levels of the active YAP protein (Supplementary Fig. 4c). On the other hand, the expression of TEAD4 clearly induced the mRNA levels of CTGF and *BCL2L1* (Supplementary Fig. 4d) and the protein expression of BCL-xL (Supplementary Fig. 4e). It is also noteworthy that a recent study demonstrated that high YAP activity in culture adapted or genetically variant hPSCs leads to beating the competition against normal hPSCs for further clonal dominance in culture^38^.

### Aurora A stabilizes the YAP1 protein

**Fig. 5:**
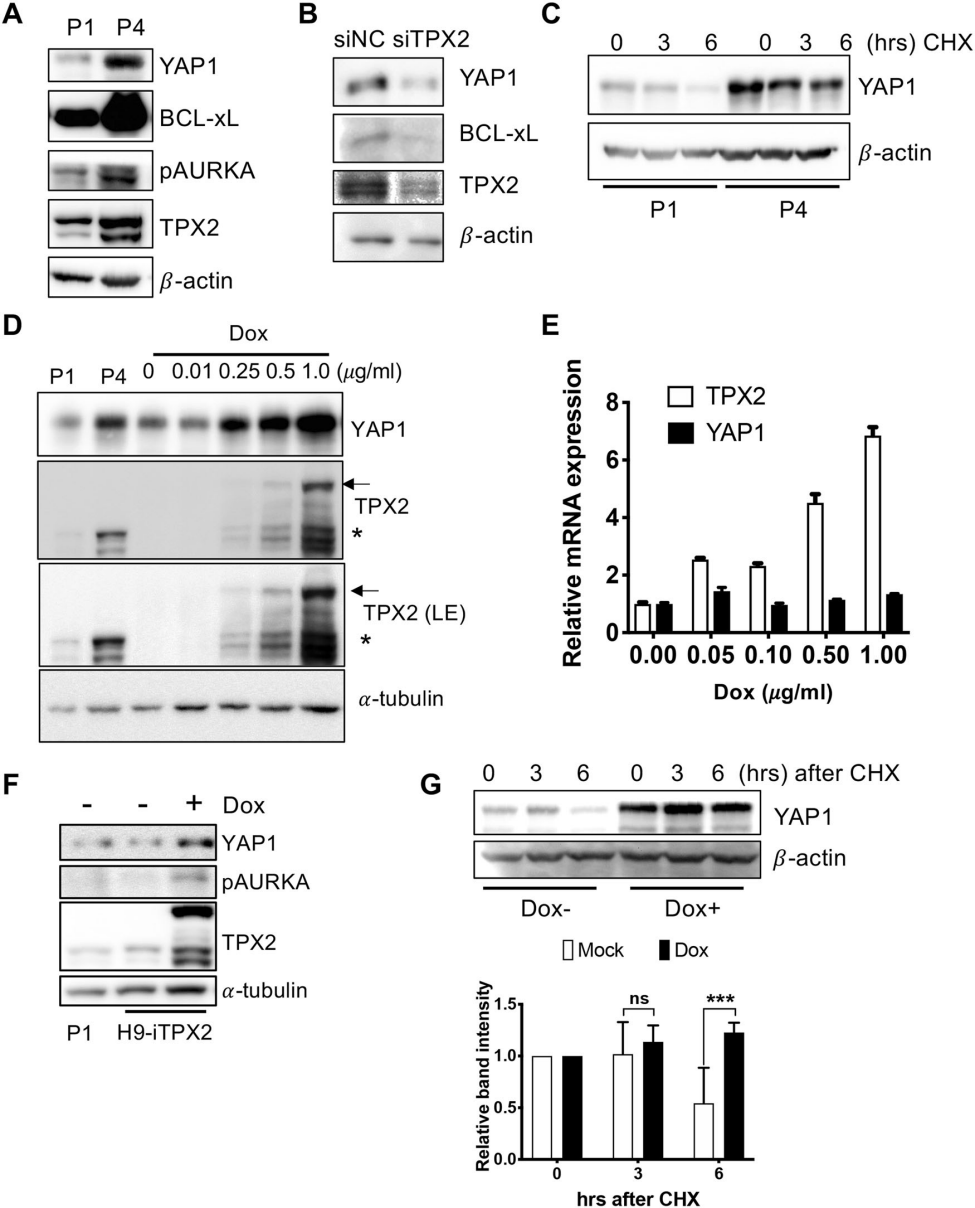
Aurora A stabilizes the YAP1 protein. **A** Immunoblotting of YAP1, BCL-xL, TPX2, and phospho-Aurora A (pAURKA) in P1 and P4 hESCs. β-Actin was used as a loading control for equal protein concentrations. **B** Immunoblotting of YAP1, BCL-xL, and TPX2 in P4 hESCs 2 days after the introduction of control (siNC) and TPX2 (siTPX2) siRNA. β-Actin was used as a loading control for equal protein concentrations. **C** Immunoblotting of YAP1 in P1 or P4 hESCs after 200 μg/ml cycloheximide (CHX) treatment, with β-actin as a loading control for equal protein concentrations. **D** Immunoblotting for YAP1 or TPX2 after treatment with the indicated dose of Dox for 24 h (arrow for induced GFP-TPX2, * for endogenous TPX2). α-Tubulin was utilized for equal protein loading. **E** The relative levels of TPX2 or YAP1 mRNA in iTPX2-hESCs after treatment with the indicated dose of Dox for 24 h. **F** Immunoblotting of YAP1, TPX2, and phospho-Aurora A (pAURKA) after 24 h treatment with 0.1 μg/ml Dox. **G** Immunoblotting of YAP1 in iTPX2-hESCs with or without 0.1 μg/ml Dox for the indicated time after treatment with 200 μg/ml CHX. β-Actin was utilized as a loading control for equal protein concentrations (top). Graph of the normalized band intensity (*n* = 4 independent experiments; mean ± SEM, two-way ANOVA, ***p < 0.001, bottom).

Previously, Aurora A, a mitotic kinase strongly associated with TPX2 in spindle assembly, was shown to phosphorylate and stabilize YAP1 in cancer^9,39^. Constitutive activation of Aurora A, which corresponds with TPX2 induction regardless of cell cycle phase, was a distinct cellular phenotype of P3 and P4 hESCs^17^. P4 hESCs with high Aurora A activity (determined by phospho-Aurora A) and TPX2 protein levels exhibited higher protein levels of BCL-xL and YAP1 (Fig. 5A) than P1 and P2 hESCs (Supplementary Fig. 5a). Of note, P3 and P4 hESCs with higher Aurora A activation at a similar level of mitotic population (determined by the level of phospho-histone H3, pHH3) along with high TPX2 under mitotic stress were evidently resistant to Noc-induced cell death (Supplementary Fig. 5b). Consistently, transient depletion of TPX2 lowered the protein levels of BCL-xL and YAP1 (Fig. 5B). Next, to confirm that the increased YAP1 protein level results from protein stability, the YAP1 protein was monitored after cycloheximide (CHX) treatment to inhibit protein translation in P1 and P4 hESCs (Fig. 5C). Similarly, the increased level of the YAP1 protein was evident in Dox-dependent TPX2 induction (Fig. 5D), whereas the YAP1 mRNA level remained unaffected (Fig. 5E). Aurora A active phosphorylation was clearly induced by TPX2 induction in iTPX2-hESCs, which was concurrent with the increase in YAP1 protein expression (Fig. 5F). Similar to P4 hESCs, the YAP1 protein but not TEAD4 (Supplementary Fig. 5c) was highly stabilized after Dox treatment in iTPX2-hESCs (Fig. 5G). These data suggest that high YAP1 protein levels in culture-adapted hESCs result from high Aurora A activity induced by TPX2.

### Inhibition of Aurora A abrogates the resistance to mitotic stress by YAP1 destabilization

**Fig. 6:**
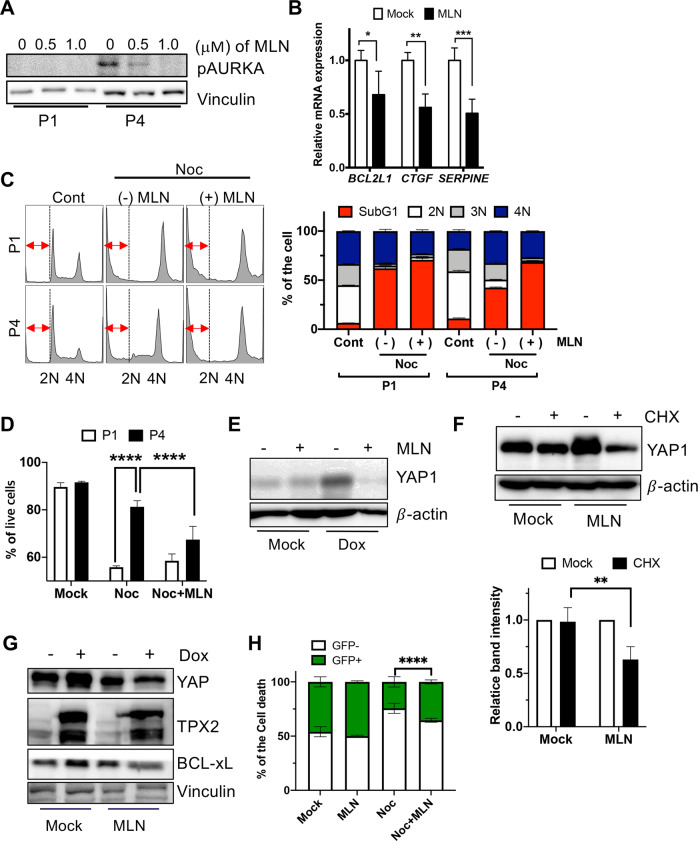
Inhibition of Aurora A abrogates resistance to mitotic stress by YAP1 destabilization. **A** Immunoblotting for phospho-Aurora A (pAURKA) after treatment with the indicated dose of MLN8237 (MLN) in P1 or P4 hESCs. Vinculin was used as the loading control. **B** Relative mRNA levels in P4 hESCs after 24 h of treatment with 0.5 μM MLN (*n* = 4 independent experiments; mean ± SEM, two-way ANOVA, *p < 0.05, **p < 0.01, ***p < 0.001). **C** Flow cytometry analysis of DNA contents. The sub-G1 population (red arrow) is presented after 24 h of treatment with nocodazole (Noc: 50 ng/ml) with or without 0.5 μM MLN (left). Graphical presentation of each phase of the cell cycle (right). **D** Percentages of live cells quantified by flow cytometry with Annexin V/7-AAD staining. After 4 h of pretreatment with MLN, the cells were treated with 50 ng/ml nocodazole with or without 0.5 µM MLN for 24 h (*n* = 6 independent experiments; mean ± SEM, two-way ANOVA, ****p < 0.0001). **E** Immunoblotting for YAP1 in iTPX2-hESCs. After 24 h of treatment with 0.1 μg/ml Dox, 0.5 μM MLN was added for 24 h of treatment with or without Dox. β-Actin was used as an equal loading control. **F** Immunoblotting for YAP1 in iTPX2-hESCs. YAP1 protein levels after 6 h after treatment with CHX (induction with 0.1 μg/ml TPX2 for 24 h prior to the experiment and then pretreatment with 0.5 μM MLN occurred 1 h prior to CHX treatment). The normalized YAP1 protein band intensity is shown (*n* = 3 independent experiments; mean ± SEM, two-way ANOVA, **p < 0.01). **G** Immunoblotting for YAP1 or BCL-xL in iTPX2-hESCs 24 h after treatment with 0.5 µM MLN. **H** The relative of GFP-positive or GFP-negative live iTPX2-hESC populations (*n* = 4 independent experiments; mean ± SEM, two-way ANOVA. ****p < 0.000).

Given that the activity of Aurora A in hESCs with high TPX2 expression appeared to stabilize YAP1, we next tested whether chemical inhibition of Aurora A would destabilize YAP1 and sensitize hESCs with high TPX2 expression to mitotic stress. To this end, we first determined the appropriate concentration of an Aurora A inhibitor (MLN8237: MLN) to inhibit Aurora A in hESCs. Intriguingly, Aurora A activity determined by its active phosphorylation was significantly reduced by 50 nM MLN treatment in P1 hESCs, whereas Aurora A remained active in P4 hESCs with up to 100 nM MLN (Supplementary Fig. 6a). Clear attenuation of active phosphorylation in P4 hESCs was distinct from treatment with 0.5 μM MLN (Fig. 6A). Therefore, all downstream experiments for P4 survival were performed using 0.5 μM MLN. In parallel with Aurora A inhibition by MLN (Fig. 6A), *BCL2L1* transcription was reduced along with *CTGF* and *SERPINE1*, which are downstream genes of YAP-TEAD4^35,40^ (Fig. 6B). Mitotic resistance in P4 hESCs, as determined by the sub-G1 populations, was also significantly attenuated by additional MLN treatment with Noc (Fig. 6C). The same result was obtained by flow cytometry analysis to determine the live cell population (Fig. 6D). Consistently, marked stabilization of YAP1 by Dox in iTPX2-hESCs was reversed by MLN treatment (Fig. 6E). The reduction in the YAP1 protein by MLN resulted from YAP1 protein destabilization by MLN (Fig. 6F), which further led to downregulation of the BCL-xL protein (Fig. 6G). Accordingly, MLN treatment significantly sensitized the GFP-positive iTPX2-hESC population to mitotic stress (Fig. 6H). In addition, treating P4 hESCs with LY3295668 (LY), a specific inhibitor of Aurora A^41^, markedly attenuated the level of active YAP1 (Supplementary Fig. 6b), and the BCL-xL protein sensitized P4 hESCs to mitotic stress (Supplementary Fig. 6c, d).

## Discussion

Despite the widespread concerns regarding the frequent genetic aberrations and consequently acquired survival trait of hESCs^3^, the molecular mechanisms underlying such drastic phenotypic changes have not been fully determined, with the exception of the known role of *BCL2L1* induction followed by 20q11.21 CNV^8,10,11^. Here, we demonstrated that *BCL2L1* transcription was induced by YAP1 stabilization due to high Aurora A activity via TPX2 induction at locus 20q11.21. Furthermore, *BCL2L1* induction (Fig. 2) followed by YAP1 protein stabilization (Fig. 5) was readily achieved by TPX2 induction without 20q11.21 CNV (Fig. 2I), resulting in survival under mitotic stress (Fig. 3). In addition, human iPSCs with 20q11.21 CNV occurring in a relatively early passage (passage 30) failed to acquire the survival trait (data not shown), whereas late-passage iPSCs (e.g., passage 180) and gain of 20q11.21 (Supplementary Fig. 1d) with high *TPX2* and *BCL2L1* expression (Supplementary Fig. 1e), showed evident survival traits^8^. Therefore, it was readily surmised that additional event(s) would occur to trigger *BCL2L1* expression in hPSCs with 20q11.21 CNV to gain survival traits.

This result is consistent with a recent study showing that human iPSCs with 20q11.21 CNV only have lesser commitment of the ectodermal lineage in teratoma with minor changes in the expression of genes in 20q11.21 loci^42^. Therefore, we propose that other cues to activate *BCL2L1* promoter activity, such as YAP1 activation, would occur, leading to the acquisition of the survival advantage trait (e.g., YAP1 stabilization by Aurora A activity due to TPX2 induction). A recent study demonstrated that the Hedgehog signal is responsible for TPX2 induction through the FOXM1 transcription factor in cancer cells^30^. Considering the complexity of the YAP1 activation mechanism, we could not rule out the role of other events [e.g., F-actin stabilization^25^] in YAP1 activation to induce *BCL2L1* expression for culture adaptation of hESCs other than the TPX2-Aurora A axis.

TPX2, with basal expression that is higher in normal hESCs than in differentiated cells^17^, was critical for the self-renewal of hESCs, as stable depletion of TPX2 failed to be achieved after multiple attempts (data not shown). Thus, only transient depletion of TPX2 with two independent siRNA sequences was performed. These results further support that TPX2 knockout leads to the failure of early embryogenesis prior to blastocyst formation^32^. As TPX2 induction stabilizes the mitotic spindle and leads to abnormal mitosis^17^, TPX2 induction occurring in multiple culture-adapted cell lines would serve as a potential driver for not only acquired survival but also further genetic alterations by saving hESCs from cell death after abnormal mitosis.

Unlike other somatic cells, hESCs are highly sensitive to genotoxic stress, which is believed to be a major safeguard system to ensure genome integrity^43^. This extremely high susceptibility to DNA damage is mostly triggered by high mitochondrial priming to apoptosis^16^ through p53 mitochondrial translocation^13^. Thus, p53 dominant-negative mutation^12^, *NOXA* mutation^9^, and *BCL2L1* induction^10^ abrogate the unique genome safeguard mechanism of high mitochondrial priming, which is commonly observed in culture-adapted hESCs. Therefore, the incidence of random mutations, including CNV at 20q11.21 (where *TPX2* and *BCL2L1* are located), during prolonged culture favors survival via TPX2 induction to activate YAP1-dependent *BCL2L1* induction in hESCs with 20q11.21 CNV would be sufficient for dominant selection during multiple bottleneck events^44^. Thus, continuous loss of high mitochondrial priming and building resistance to mitotic stress may collectively enhance chromosome instability and even induce aneuploidy during long-term hESC culture.

## Supplementary information

Supplemental Materials

## Data Availability

Source data are available from the corresponding author upon request. The RNA-seq results have been deposited in the Gene Expression Omnibus (GEO) under accession number GSE 167495. The flow cytometry data were deposited to https://flowrepository.org/id/FR-FCM-Z435.

## References

[1] Lund RJ, Narva E, Lahesmaa R (2012). Genetic and epigenetic stability of human pluripotent stem cells. Nat. Rev. Genet..

[2] Heslop JA (2015). Concise review: workshop review: understanding and assessing the risks of stem cell-based therapies. Stem Cells Transl. Med..

[3] Andrews PW (2017). Assessing the Safety of Human Pluripotent Stem Cells and Their Derivatives for Clinical Applications. Stem Cell Rep..

[4] Halliwell J, Barbaric I, Andrews PW (2020). Acquired genetic changes in human pluripotent stem cells: origins and consequences. Nat. Rev. Mol. Cell Biol..

[5] Baker DE (2007). Adaptation to culture of human embryonic stem cells and oncogenesis in vivo. Nat. Biotechnol..

[6] Spits C (2008). Recurrent chromosomal abnormalities in human embryonic stem cells. Nat. Biotechnol..

[7] Lefort N (2008). Human embryonic stem cells reveal recurrent genomic instability at 20q11.21. Nat. Biotechnol..

[8] Cho, S. J. et al. Selective Elimination of Culture-Adapted Human Embryonic Stem Cells with BH3 Mimetics. *Stem Cell Rep*. **11**, 1–13 (2018). 10.1016/j.stemcr.2018.09.002 PMC6235677 30293852

[9] Zhang J (2019). Anti-apoptotic Mutations Desensitize Human Pluripotent Stem Cells to Mitotic Stress and Enable Aneuploid Cell Survival. Stem Cell Rep..

[10] Avery S (2013). BCL-XL mediates the strong selective advantage of a 20q11.21 amplification commonly found in human embryonic stem cell cultures. Stem Cell Rep..

[11] Nguyen HT (2014). Gain of 20q11.21 in human embryonic stem cells improves cell survival by increased expression of Bcl-xL. Mol. Hum. Reprod..

[12] Merkle FT (2017). Human pluripotent stem cells recurrently acquire and expand dominant negative P53 mutations. Nature.

[13] Lee MO (2013). Inhibition of pluripotent stem cell-derived teratoma formation by small molecules. Proc. Natl Acad. Sci. USA.

[14] TeSlaa T, Setoguchi K, Teitell MA (2016). Mitochondria in human pluripotent stem cell apoptosis. Semin. Cell Dev. Biol..

[15] Dumitru, R. et al. Human Embryonic Stem Cells Have Constitutively Active Bax at the Golgi and Are Primed to Undergo Rapid Apoptosis. *Mol. Cell.* **46**, 573–583 (2012). 10.1016/j.molcel.2012.04.002 PMC3372694 22560721

[16] Liu JC (2013). High mitochondrial priming sensitizes hESCs to DNA-damage-induced apoptosis. Cell Stem Cell.

[17] Jeong, H.-C. et al. TPX2 Amplification-Driven Aberrant Mitosis in Long-Term Cultured Human Embryonic Stem Cells. *bioRxiv*10.1101/2021.02.22.432205 (2021).

[18] Lamm N (2016). Genomic Instability in Human Pluripotent Stem Cells Arises from Replicative Stress and Chromosome Condensation Defects. Cell Stem Cell.

[19] Thompson O (2020). Low rates of mutation in clinical grade human pluripotent stem cells under different culture conditions. Nat. Commun..

[20] Weissbein U, Benvenisty N, Ben-David U (2014). Quality control: Genome maintenance in pluripotent stem cells. J. Cell Biol..

[21] Liu JC, Lerou PH, Lahav G (2014). Stem cells: balancing resistance and sensitivity to DNA damage. Trends Cell Biol..

[22] Morin-Kensicki EM (2006). Defects in Yolk Sac Vasculogenesis, Chorioallantoic Fusion, and Embryonic Axis Elongation in Mice with Targeted Disruption of Yap65. Mol. Cell Biol..

[23] Lian I (2010). The role of YAP transcription coactivator in regulating stem cell self-renewal and differentiation. Genes Dev..

[24] Leblanc L (2018). Yap1 safeguards mouse embryonic stem cells from excessive apoptosis during differentiation. eLife.

[25] Ohgushi, M., Minaguchi, M. & Sasai, Y. Rho-Signaling-Directed YAP/TAZ Activity Underlies the Long-Term Survival and Expansion of Human Embryonic Stem Cells. *Cell Stem Cell* **17**, 448–461 (2015). 10.1016/j.stem.2015.07.009 26321201

[26] Rosenbluh J (2012). β-Catenin-driven cancers require a YAP1 transcriptional complex for survival and tumorigenesis. Cell.

[27] Kim KT (2020). Safe scarless cassette-free selection of genome-edited human pluripotent stem cells using temporary drug resistance. Biomaterials.

[28] Go YH (2019). Structure-Activity Relationship Analysis of YM155 for Inducing Selective Cell Death of Human Pluripotent Stem Cells. Front. Chem..

[29] Markouli C (2019). Gain of 20q11.21 in Human Pluripotent Stem Cells Impairs TGF-β-Dependent Neuroectodermal Commitment. Stem Cell Rep..

[30] Wang Y (2020). The critical role of dysregulated Hh-FOXM1-TPX2 signaling in human hepatocellular carcinoma cell proliferation. Cell Commun. Signal..

[31] Warner SL (2009). Validation of TPX2 as a potential therapeutic target in pancreatic cancer cells. Clin. Cancer Res..

[32] Aguirre-Portoles C (2012). Tpx2 controls spindle integrity, genome stability, and tumor development. Cancer Res..

[33] Reid TA (2016). Suppression of microtubule assembly kinetics by the mitotic protein TPX2. J. Cell Sci..

[34] Meyer-Ficca ML (2004). Comparative analysis of inducible expression systems in transient transfection studies. Anal. Biochem..

[35] Zhao B (2008). TEAD mediates YAP-dependent gene induction and growth control. Genes Dev..

[36] Hansen CG, Moroishi T, Guan KL (2015). YAP and TAZ: a nexus for Hippo signaling and beyond. Trends Cell Biol..

[37] Guo X (2017). Single tumor-initiating cells evade immune clearance by recruiting type II macrophages. Genes Dev..

[38] Price CJ (2021). Genetically variant human pluripotent stem cells selectively eliminate wild-type counterparts through YAP-mediated cell competition. Dev. Cell.

[39] Chang SS (2017). Aurora A kinase activates YAP signaling in triple-negative breast cancer. Oncogene.

[40] Choi JY (2021). TGFbeta promotes YAP-dependent AXL induction in mesenchymal-type lung cancer cells. Mol. Oncol..

[41] Du J (2019). Aurora A-Selective Inhibitor LY3295668 Leads to Dominant Mitotic Arrest, Apoptosis in Cancer Cells, and Shows Potent Preclinical Antitumor Efficacy. Mol. Cancer Ther..

[42] Jo HY (2020). Functional in vivo and in vitro effects of 20q11.21 genetic aberrations on hPSC differentiation. Sci. Rep..

[43] Weissbein U, Benvenisty N, Ben-David U (2014). Genome maintenance in pluripotent stem cells. J. Cell Biol..

[44] Barbaric I (2014). Time-lapse analysis of human embryonic stem cells reveals multiple bottlenecks restricting colony formation and their relief upon culture adaptation. Stem Cell Rep..

