# Supplementary material for: TPX2 prompts mitotic survival via the induction of BCL2L1 through YAP1 protein stabilization in human embryonic stem cells
Source: Exp Mol Med. 2023 Jan 4;55(1):32–42. doi: 10.1038/s12276-022-00907-9 (PMC9898288; doi:10.1038/s12276-022-00907-9)

TPX2 prompts mitotic survival via the induction of *BCL2L1* through YAP1 protein stabilization in human embryonic stem cells

Running Title: TPX2 for hESC mitotic survival

Yun-Jeong Kim[1], Young-Hyun Go[2], Ho-Chang Jeong[2], Eun-Ji Kwon[1], Seong-Min Kim[1], Hyun Sub Cheong[3], Wantae Kim[4], Hyoung Doo Shin[2], Haeseung Lee[5], and Hyuk-Jin Cha[1#]

This PDF file includes:

Supplementary Materials and Methods

Supplementary Tables

Supplementary Figure legends

Supplementary Figures 1-6

Supplementary Materials and Methods

Immunoblotting

Cell lysates were extracted with RIPA buffer supplemented with a 1% protease inhibitor cocktail and 0.1% sodium orthovanadate. After a 1 hour incubation on ice, total protein was extracted by centrifugation. The concentration of total protein was quantified by a BCA protein assay kit (#23225, Thermo ScientificTM). Approximately 15-20 µg of total protein was separated by various (7.5%, 10%, 15%) concentrations of SDS–PAGE. The separated protein in the gel was transferred to a PVDF membrane. Membranes with protein were blocked with 5% skim milk in Tris-buffered saline with 0.1% Tween-20 (TBS-T) for 1 hour and then washed with TBS-T three times (5 minutes each time). The membrane was incubated with the primary antibody in TBS-T (1:1000) with 0.1% sodium azide overnight at 4 °C. The incubated membrane was washed three times (5 minutes each time) with TBS-T. The membrane was incubated with HRP-conjugated secondary antibody (Jackson Immunoresearch Laboratories) in TBS-T (1:10000) for 1 hour at room temperature. The incubated membrane was then washed three times (15 minutes each time) with TBS-T. Immunoreactivity was detected by ChemiDoc using a WEST-QueenTM kit (#16026, iNtRON Biotechnology). The band intensity was measured using Fusion FX software and normalized to the loading control.

Dual-luciferase assay

Cells were transfected with a specific promoter-luciferase vector (8X GTIIC luciferase reporter vector (kindly gifted by Professor Mo Jung-Soon at Ajou University)) and pRL Renilla luciferase control reporter vector (cat. no. E223A; Promega Corporation). Cell lysates were extracted with 1X passive lysis buffer. After a 1 hour incubation on ice, the total lysate was extracted by centrifugation. The supernatant was used for reaction with LARII and Stop & Glo

reagent. The reporter assay was performed according to the Dual-Luciferase Reporter Assay System (#E1980, Promega).

Cell death assay

Cell death was analyzed by flow cytometry. For Annexin V/7-AAD staining, cells 24 h after treatment with each flavonoid were washed twice with PBS and stained with FITC-conjugated Annexin V antibody (BD Bioscience, Franklin Lakes, NJ, USA, #556419) and 7-AAD (BD Bioscience, #559925), PE Annexin V antibody (BD Bioscience, 556421) or propidium iodide (PI) for an additional 45–60 min at room temperature in the dark. Cells stained with Annexin V/7-AAD were analyzed by FACS Calibur (BD Bioscience). To capture the bright field images, a light channel optical microscope (Olympus, Tokyo, Japan, CKX-41) or JULI-stage (NanoEntek, Seoul, Korea) was used in accordance with the manufacturer's protocol.

RT-qPCR analysis

Easy-BLUE^{TM} RNA isolation kit (iNtRON Biotechnology) is used for total RNA extraction. PrimeScript^{TM} RT reagent kit (TaKaRa) is used to generate cDNA from RNA extracted previously. Quantitative real-time PCR analysis was performed with QuantStudiot^{TM} (Applied Biosystems) and SYBR® Green PCR reagents (Life Technologies) are used for quantitative real-time PCR analysis, following the supplier’s instructions. Primers and sequences for siRNAs are listed in Supplementary Table 4 and 5.

Supplementary Table 1. Antibody information

Antibody	Catalog	Company
α-tubulin	#sc- 8035	Santa Cruz Biotechnology
β-actin	#sc-47778	Santa Cruz Biotechnology
YAP1/TAZ	#sc-101199	Santa Cruz Biotechnology
Vinculin	#sc-25336	Santa Cruz Biotechnology
eGFP	#sc-9996	Santa Cruz Biotechnology
TEAD4	#sc-101199	Santa Cruz Biotechnology
phospho-YAP1	#4911s	Cell Signaling Technology
phospho-Aurora A	#3079	Cell Signaling Technology
TPX2	#12245	Cell Signaling Technology
active YAP antibody	#ab205270	Abcam
BCL-xL	#ab32370	Abcam

Supplementary Table 2. Chemical information

Chemical	Catalog	Company
MLN8237	#S1133	Selleckchem
Nocodazole	#R17934	Selleckchem
LY3295668	#HY-114258	MedChemExpress
Matrigel	#354277	Corning
Gentamycin	#15750-060	Gibco

Supplementary Table 3. Cell culture

Name	Catalog	Company
iPSC-brew	#130-104-368	Miltenyi Biotechnology
Dispase	#11320-041 5g	Life technology
DMEM/F12	#11320-033	Gibco
Y27632	#1293823	PeproTech

Supplementary Table 4. RT-qPCR primer sequence

Gene symbol	Primer sequence (5' to 3')
18srRNA	F: GTA ACC CGT TGA ACC CCA TT<br>R: CCA TCC AAT CGG TAG TAG CG
GAPDH	F: AAG GGT CAT CAT CTC TGC CC<br>R: GTC ATG GCA TGG ACT GTG GT
ACTB	F: GTC CTC TCC CAA GTC CAC AC<br>R: GGG AGA CCA AAA GCC TTC AT

TPX2	F: GCT CAA CCT GTG CCA CAT TA<br>R: CGA GAA AGG GCA TAT TTC CA
GFP	F: CCG GAC CTC CAA AGA AAA A<br>R: AAA AGT GAC CCC CGA CCT T
BCL2L1	F: GAT CCC CAT GGC AGC AGT AAA GCA AG<br>R: CCC CAT CCC GGA AGA GTT CAT TCA CT
BIRC5	F: GGA CCA CCG CAT CTC TAC<br>R: GCA CTT TCT TCG CAG TTT
YAP	F: GTG AGC CTG TTT GGA TGA TG<br>R: CAC TGG ACA AAG GAA GCT GA
TAZ	F: CCA GGT GCT GGA AAA AGA AG<br>R: CAG GAT GAT GGG GTT GAG AT
CTGF	F: CCA ATG ACA ACG CCT CCT G<br>R: TGG TGC AGC CAG AAA GCT C
SERPINE1	F: TTG AAT CCC ATA GCT GCT TGA AT<br>R: ACC GCA ACG TGG TTT TCT CA
TEAD4	F: GAA CGG GGA CCC TCC AAT G<br>R: GCG AGC ATA CTC TGT CTC AAC

Supplementary Table 5. siRNA sequence

Gene Symbol	siRNA sequence (5' to 3')
TPX2 #3	S: CAG GAU UUU GCU GUG AAG U<br>AS: ACU UCA CAG CAA AAU CCU G
TPX2 #4	S: AGC AAG UUG AAG ACU UCC AUA<br>AS: UAU GGA AGU CUU CAA CUU GCU
TEAD4	S: CCG CCA AAU CUA UGA CAA ATT<br>AS: UUU GUC AUA GAU UUG GCG GTT
YAP	S: CAG AAG AUC AAA GCU ACU U<br>AS: AAG UAG CUU UGA UCU UCU G
TAZ	S: ACG UUG ACU UAG GAA CUU U<br>AS: AAA GTT CCT AAG TCA ACG T

Supplementary Figure Legends

Supplementary Fig. 1. (a) Cell death analysis of H9 under 50ug/ml of YM155 for 24hr in passage dependent manner (left), Bar graph for % of live cells (right) **(b)** Images of clonogenic assay of P1, P2, P3 and P4 hESCs, 48 hours after single cell dissociation in the absence (Mock) or treatment of Y27632 (left), Bar graph of relative area (A.U) of clonogenic assay (right, n=3 independent experiments; mean ± SEM, One-Way ANOVA, **p<0.01) **(c)** Bar graph of % of live cells of P1, P2, P3 and P4 hESCs after 24hrs of 50ng/ml Nocodazole(Noc) treatment. **(d)** Copy number output using the Illumina Asian Screening Array (~700K) as computed by GenomeStudio software provided by Illumina. The two plots shown are for B allele frequency and log R ratio. The copy number gain on chromosome 20 detected in BJ-iPSCs p52/p181 and CHA3 hESC p90/p333 based on log R ratio is shown by a red box. **(e)** Relative mRNA expression of *TPX2* and *BCL2L1* in BJ-iPSCs and hCHA3 with or without 20q.11.21 CNV was represented.

Supplementary Fig. 2. (a) Relative mRNA level of *TPX2* and *BCL2L1* in P3 hESCs treated with siRNA control (siNC) and siRNA for TPX2 (siTPX2). **(b)** mRNA expression of *TPX2* and *BCL2L1* of P4 hESCs with siRNA control (siNC) and siRNA for TPX2 (siTPX2) with indicated reference genes (n=3 independent experiments; mean±SEM, 2-Way ANOVA, **p<0.01, ****p<0.0001). **(c)** mRNA expression of *BCL2L1* in P1, P2, P3 and P4 hESCs with indicated reference genes **(d)** Live cell image was conducted by JULI after dox dose dependent treatment for 24hr in iTPX2 hESCs, Right panel presents mRNA expression level of TPX2 after Dox 24hr treatment. **(e)** Immunoblotting for TPX2 and eGFP in CHA3-iTPX2. **(f)** Microscopic images of TPX2 (green) during mitosis after 1μg/ml of Dox treatment for 24hrs in iTPX2 hESCs **(g)** mRNA expression level of *TPX2* and *BCL2L1* was tested in CHA3 after

1μg/ml of Dox treatment for 24hr. n=6 independent experiments; mean±SEM, Mann-Whitney test, **p<0.01, ****p<0.0001.)

Supplementary Fig. 3. (a) Immunoblotting for TPX2 in GFP positive (GFP+) and negative (GFP-) cells after treatment of 0.1μg/ml for 16hrs in iTPX2-hESCs. **(b-c)** After 16hr of Dox 0.1μg/ml treatment, Noc and Tax are treated with same dose of Dox for 24hr. Cell death of GFP positive cell was determined by PE-Annexin staining. and negative population was displayed in right panel. **(b)** Cell death under 50ng/ml of Noc and 50μg/ml of Tax was tested in hESC-iTPX2 #5. **(c)** Mitotic cell death under 50ng/ml of Noc was tested in CHA3-iTPX2 n=6 independent experiments; mean±SEM, Mann-Whitney test, **p<0.01. **(d)** Relative mRNA expression of *TPX2* in H9-P1, P4 and iTPX2. n=4 independent experiments; mean±SEM for P1, n=5 independent experiments; mean±SEM for P4 and iTPX2. One-Way ANOVA. **(e)** Transient knockdown efficacy of TPX2 in P4(n=6 independent experiments; mean±SEM, One-Way ANOVA, ***p<0.001, ****p<0.0001). **(f)** mRNA expression of *BCL2L1* and *TPX2* was tested in P4 after siTPX2 #4 and siNC treated. **(g)** Cell susceptibility under Nocodazole stress in P4 was tested after siTPX2 #4 and siNC treatment. Quantification of cell death was presented on right panel.

Supplementary Fig. 4. (a) List of up-regulated pathway in P1 hESCs based on GSEA analysis of RNA-seq data (GSE167495) **(b)** Expression of *BCL2L1* after siNC and siTEAD4 treated with indicated reference genes (n=3 independent experiments; mean ± SEM, Two-Way ANOVA, ****p<0.0001) **(c)** Immunoblotting for indicated proteins in BJ-iPSCs and CHA3, β-actin for equal protein loading **(d)** mRNA expression level of *CTGF* and *BCL2L1* after transient expression of TEAD4 (n=6 independent experiments; mean ± SEM, multiple t-test,

***p<0.001) **(e)** Immunoblotting for BCL-xL and TEAD4 after transient TEAD4 overexpression. Vinculin as loading control.

Supplementary Fig. 5. (a) Immunoblotting for indicated protein in P1, P2, P3 and P4 hESCs, Vinculin and β-actin for equal protein loading **(b)** Immunoblotting for indicated protein in P1, P3 and P4 hESCs at 24 hours after 50ng/ml of Nocodazole treatment (NOC) (T for total, C for cleaved PARP), β-actin for equal protein loading **(c)** Immunoblotting for indicated protein in iTPX2, at 24 hours of indicated dose of Dox treatment, α-tubulin for loading protein loading.

Supplementary Fig. 6. (a) Immunoblotting of phosphorylated Aurora A (pAURKA) of P1 hESCs and P4 hESCs at 24hr treatment of indicated dose of MLN8237 (MLN), β-actin and Vinculin for equal loading **(b)** (Top) Immunoblotting for indicated protein in P4 hESCs at 24 hours of indicated dose of LY3295668 (LY), β-actin for equal protein loading. (Bottom) Brightfield view of P4 hESCs after 24hours of indicated dose of LY **(c)** Clonogenic assay of P4 hESCs for 3 days in the absence (Mock) or presence of Nocodazole (Noc: 50ng/ml) at LY treatment **(d)** Relative cell growth of P4 hESCs for 24 hours at indicated condition with or without 50ng/ml of nocodazole (Noc). n=5 independent experiments; mean±SEM, 2-Way ANOVA, ***p<0.001

Supplementary Figure. 1

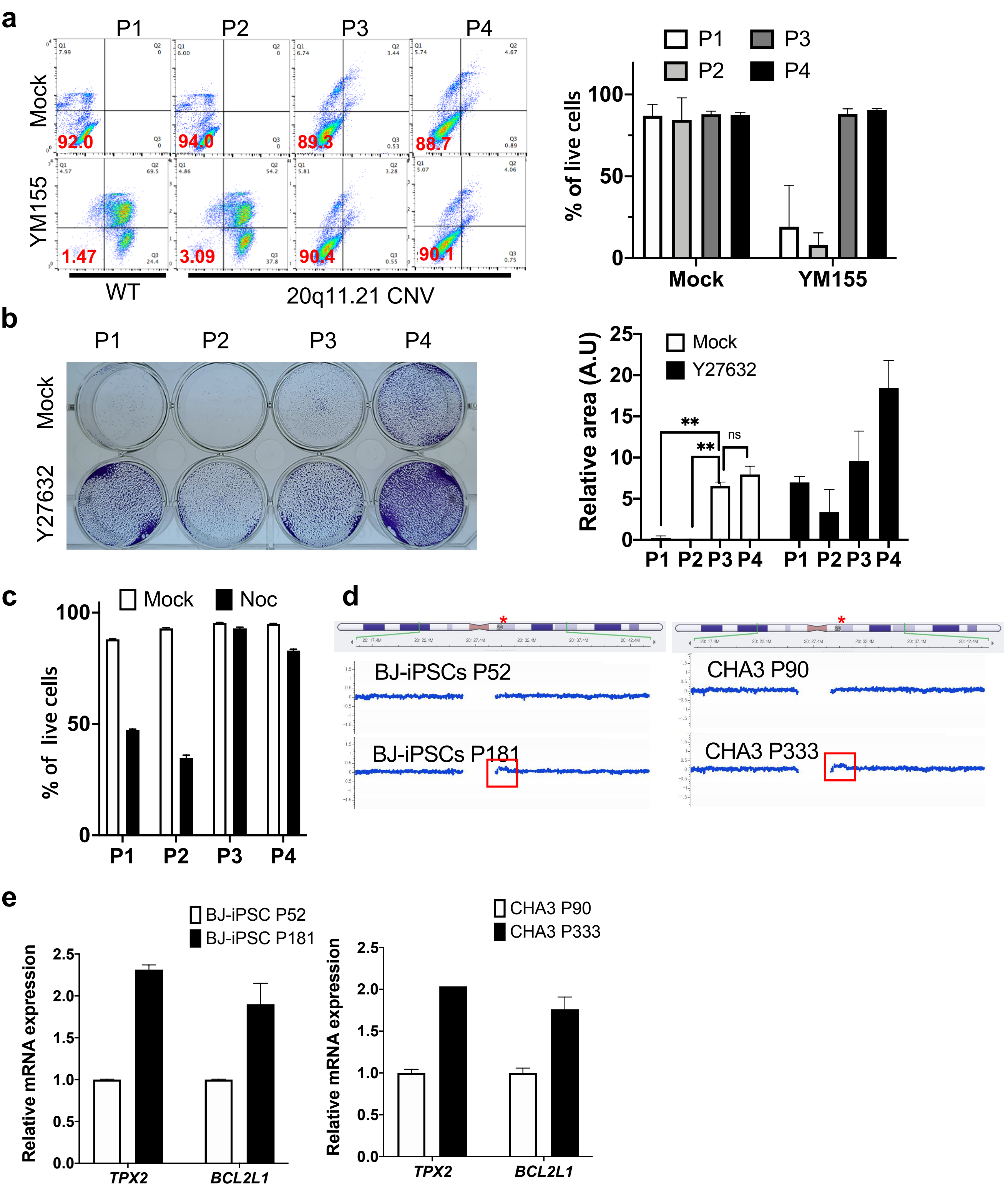

Supplementary Figure. 2

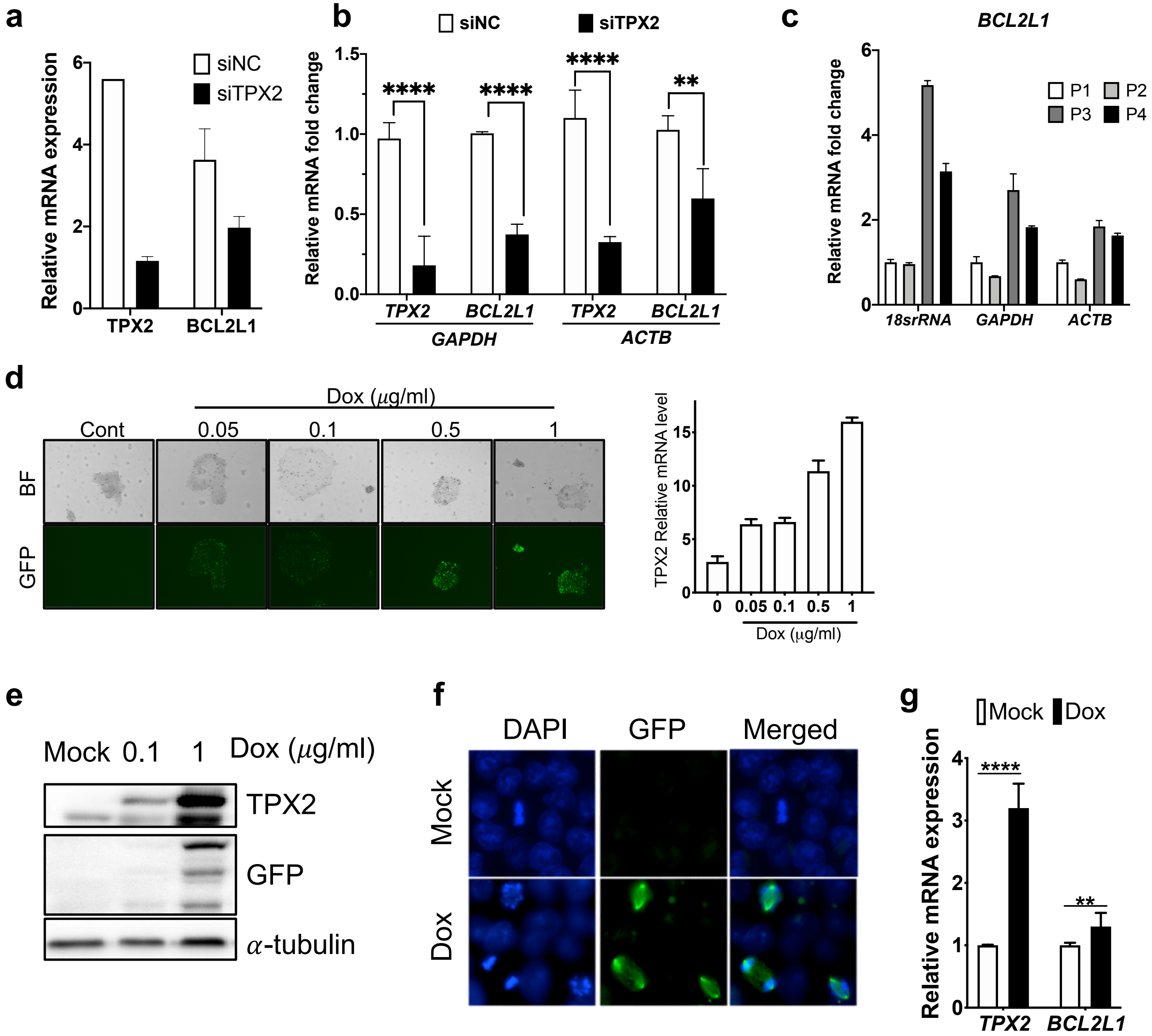

Supplementary Figure. 3

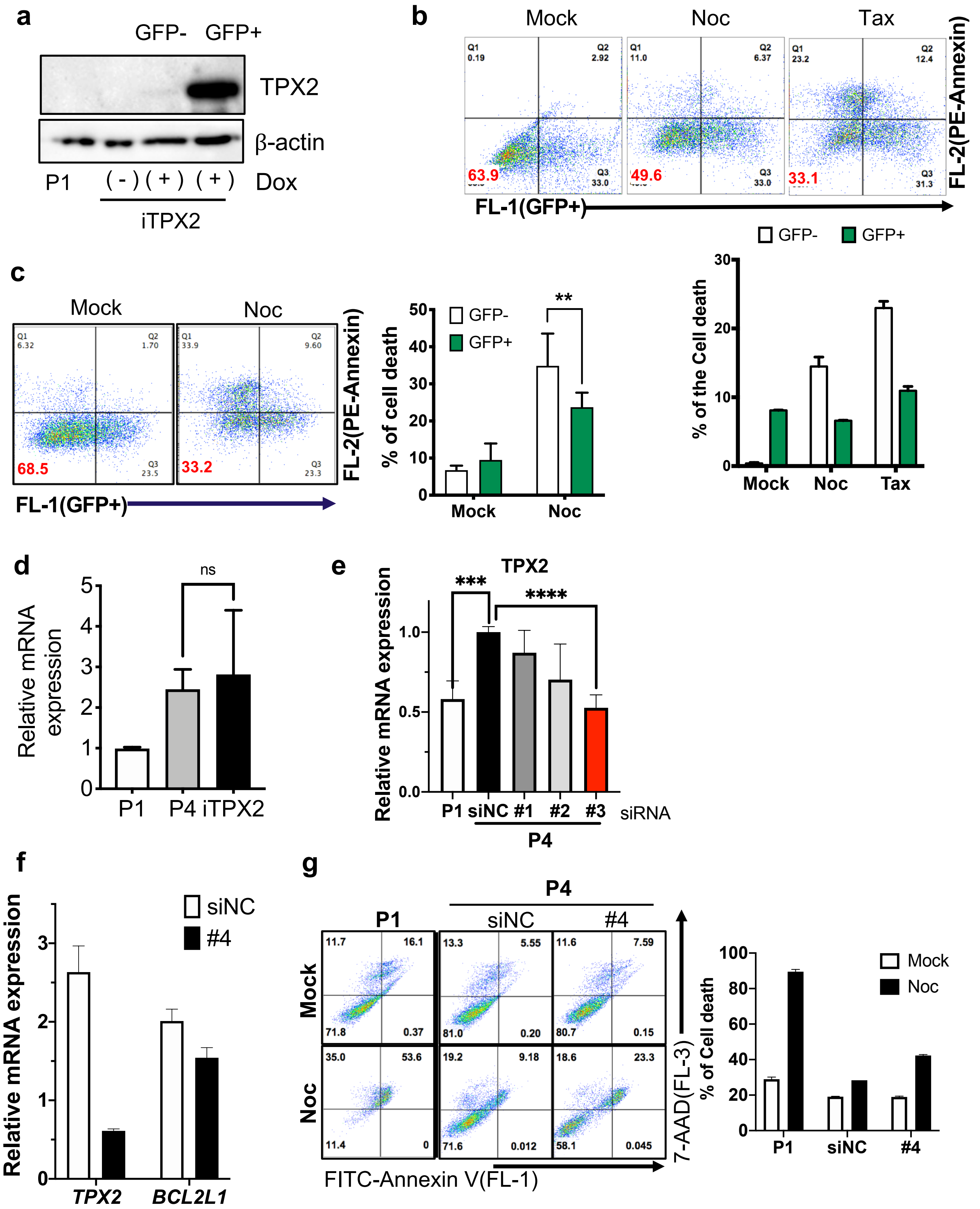

Supplementary Figure. 4

a

NAME	SIZE	ES	NES	NOM p-val	FDR q-val
WP_TGFB_SIGNALING_IN_THYROID_CELLS_FOR_EPITHELIALMESENCHYMAL_TRANSITION	18	0.72	1.71	0.00	0.09
**WP_PATHWAYS_REGULATING_HIPPO_SIGNALING**	**98**	**0.54**	**1.65**	**0.00**	**0.24**
WP_HIPPOMERLIN_SIGNALING_DYSREGULATION	120	0.53	1.64	0.00	0.19

b

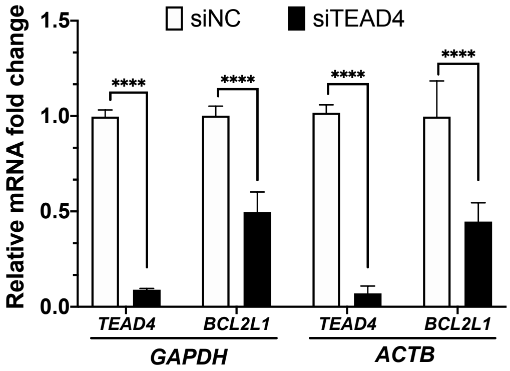

c

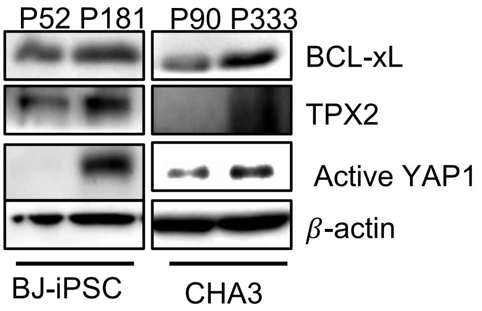

d

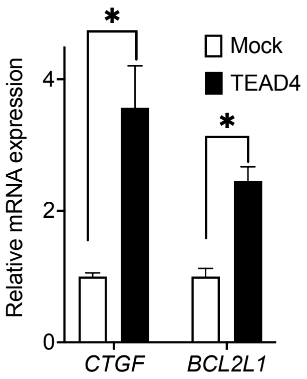

e

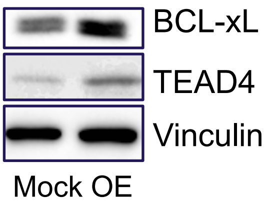

Supplementary Figure. 5

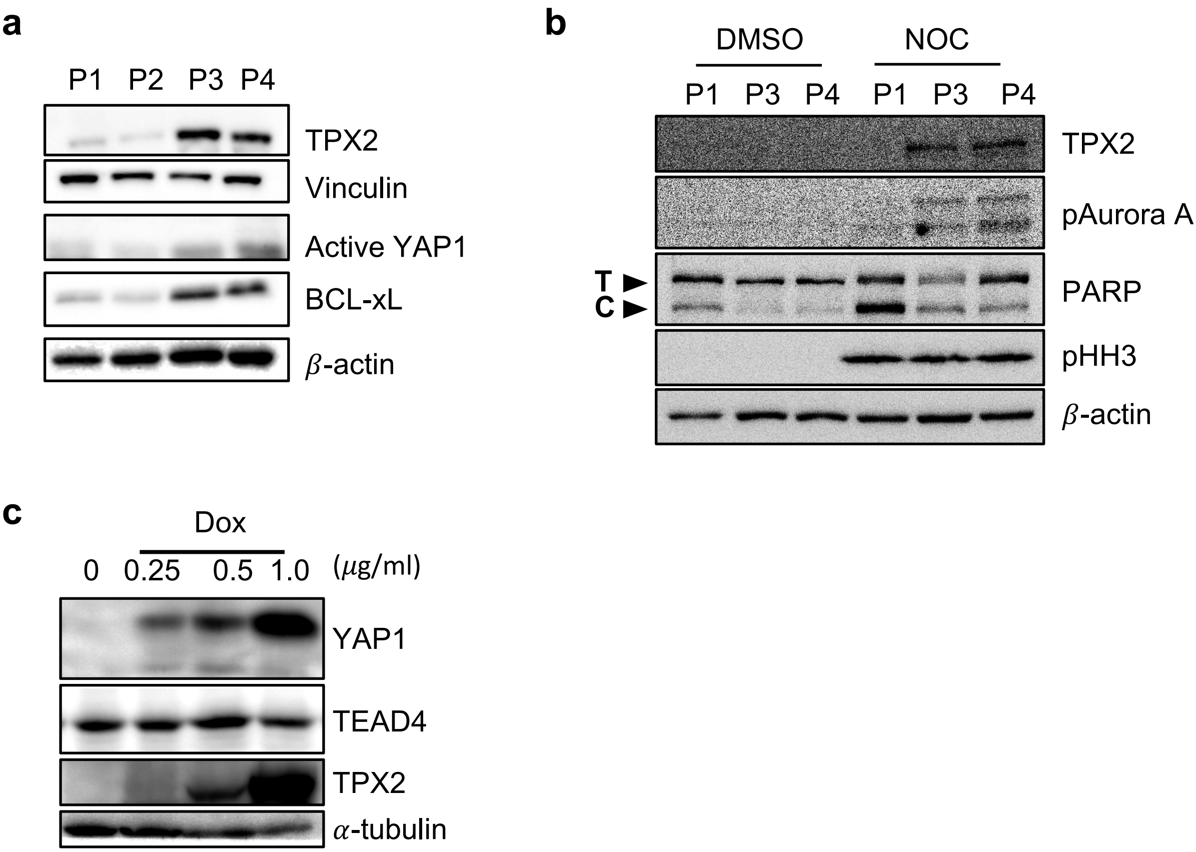

Supplementary Figure. 6

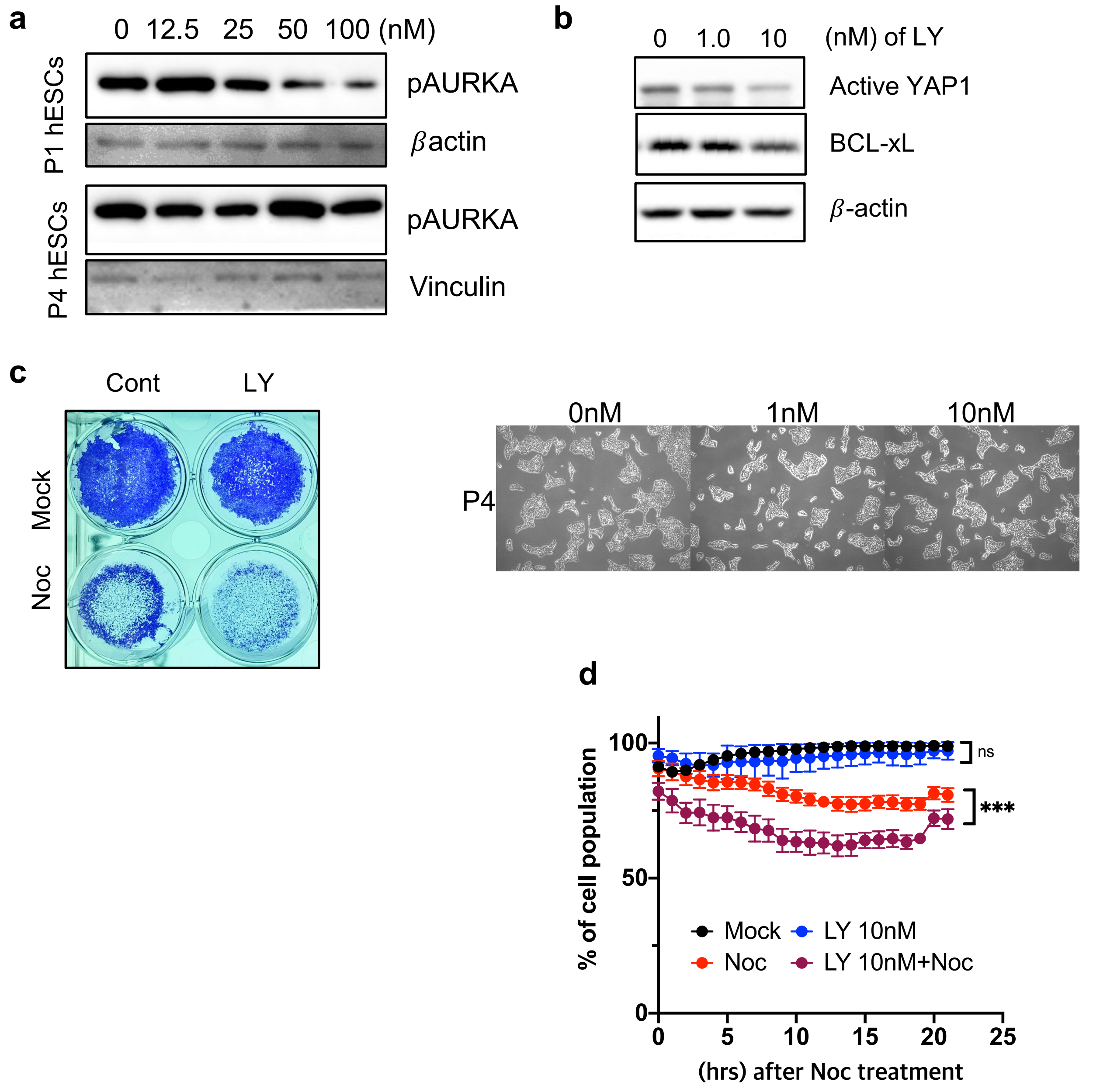

Supplement: Supplementary file 1 — Supplemental Materials [file 12276_2022_907_MOESM1_ESM.pdf]
